# Prediction of the burial status of transmembrane residues of helical membrane proteins

**DOI:** 10.1186/1471-2105-8-302

**Published:** 2007-08-20

**Authors:** Yungki Park, Sikander Hayat, Volkhard Helms

**Affiliations:** 1 Center for Bioinformatics, Saarland University, 66041 Saarbruecken, Germany

## Abstract

**Background:**

Helical membrane proteins (HMPs) play a crucial role in diverse cellular processes, yet it still remains extremely difficult to determine their structures by experimental techniques. Given this situation, it is highly desirable to develop sequence-based computational methods for predicting structural characteristics of HMPs.

**Results:**

We have developed TMX (TransMembrane eXposure), a novel method for predicting the burial status (i.e. buried in the protein structure vs. exposed to the membrane) of transmembrane (TM) residues of HMPs. TMX derives positional scores of TM residues based on their profiles and conservation indices. Then, a support vector classifier is used for predicting their burial status. Its prediction accuracy is 78.71% on a benchmark data set, representing considerable improvements over 68.67% and 71.06% of previously proposed methods. Importantly, unlike the previous methods, TMX automatically yields confidence scores for the predictions made. In addition, a feature selection incorporated in TMX reveals interesting insights into the structural organization of HMPs.

**Conclusion:**

A novel computational method, TMX, has been developed for predicting the burial status of TM residues of HMPs. Its prediction accuracy is much higher than that of previously proposed methods. It will be useful in elucidating structural characteristics of HMPs as an inexpensive, auxiliary tool. A web server for TMX is established at http://service.bioinformatik.uni-saarland.de/tmx and freely available to academic users, along with the data set used.

## Background

Helical membrane proteins (HMPs) play a crucial role in diverse cellular processes, including energy generation, signal transduction, the transport of solutes across the membrane, and the maintenance of ionic and proton concentrations. Several studies have suggested that HMPs account for 20 – 30% of the open reading frames of sequenced genomes [1,2]. In spite of their biological importance and genomic abundance, less than 1% of the proteins with known structure are HMPs [3], and this situation is not expected to improve dramatically in the near future. Hence, it is desirable to develop sequence-based computational methods for predicting structural characteristics of HMPs. In the realm of soluble proteins, two particular structural characteristics have been the main target of computational prediction methods: secondary structure [4-10] and solvent accessibility [11-26] (often in a form of binary burial status; buried inside vs. exposed to the environment). For HMPs, the prediction of secondary structures does not carry as significant a momentum as for soluble proteins because transmembrane (TM) segments, which can be relatively reliably identified from the sequence by several techniques [27-37], are known to usually adopt helical conformations to satisfy the hydrogen bonding capacity of the backbone polar atoms. On the other hand, the problem of predicting the burial status (i.e. buried in the protein core vs. exposed to the membrane) of TM residues of HMPs has remained nearly untouched until now, in contrast to the situation for soluble proteins, which have been extensively studied (see the references listed above) following the pioneering work of Rost and Sander [12]. This is quite "remarkable" given that it is much more difficult to determine the structures of HMPs than those of soluble proteins by experimental techniques. The virtue of the ability to predict the burial status of TM residues of HMPs was already appreciated by several studies around the early 90s [38-40]. The burial status prediction should be useful in several tasks. One simple example would be to help design mutational experiments aimed at identifying catalytically important TM residues [41-45] by providing a list of TM residues highly likely to be buried in the protein core because catalytically important TM residues are usually found buried in the protein core, not being exposed to the membrane. Another simple example would be to help design mutational experiments aimed at identifying TM residues important for protein-protein interactions in the membrane by providing a list of TM residues highly likely to be exposed to the membrane.

In 2004, Beuming and Weinstein pioneered the first sequence-based computational method for predicting the burial status of TM residues of HMPs (denoted hereafter as the BW method), which was based on sequence conservation patterns and a newly derived knowledge-based propensity scale of the 20 amino acids to be exposed to the membrane [46]. For a rather small benchmark set, the BW method achieved an impressive prediction accuracy of 80%. Recently, Adamian and Liang reported the development of a similar method [47], but it predicts the face of a TM helix exposed to the membrane, not the burial status of individual TM residues. Hildebrand and his coworkers described a computational method for predicting whether a given residue is located at a helix-helix interface in the membrane [48]. Yet, this is a distinct prediction problem from the one the current study deals with: a residue located outside of a helix-helix interface can still be buried. Quite recently, Yuan and his coworkers developed a method for predicting the relative solvent-accessible surface area (rSASA) of TM residues based on support vector regression (SVR, denoted hereafter as the YU method) [49]. Even though the YU method does not explicitly predict the burial status of TM residues, it is possible to do so using the predicted rSASA values. To our best knowledge, the BW and YU methods are the only ones currently available for predicting the burial status of TM residues of HMPs.

We have developed TMX (TM eXposure), a novel sequence-based computational method for predicting the burial status of TM residues of HMPs. Its accuracy is 78.71% over a much larger data set of 3138 TM residues, representing a considerable improvement over 68.67% of the BW method when evaluated on the same data set. This prediction accuracy is also higher than 71.06% of the YU method. Importantly, unlike the BW and YU methods, TMX automatically yields confidence scores for the predictions made, a highly desirable component for any computational prediction method, which allows the user to selectively utilize prediction results depending on confidence scores in real application situations. In addition, a feature selection incorporated in TMX reveals interesting insights into the structural organization of HMPs.

## Results and Discussion

### Analysis of the BW method

TMX is novel in several aspects compared to the BW and YU methods and can be described without any reference to these previous methods. However, we prefer to describe the logic behind its development in reference to the BW method in order to contrast it with the BW method and highlight its novelties.

For predicting the burial status of a TM residue, the BW method computes its positional score and compares the score with a threshold [46]. If the score is higher than the threshold, it is predicted to be buried. Otherwise, it is predicted to be exposed to the membrane. Formally, the BW method computes a positional score for sequence position *i*, *S*(*i*), as 0.5×(*C*(*i*) - *P*_*BW*_(*i*)), where *C*(*i*) is the conservation index for sequence position *i*, and *P*_*BW*_(*i*) the propensity of sequence position *i *for being exposed to the membrane, which is in turn derived from the BW scale as shown in Eq. 1. The BW scale is derived from a set of HMPs with known structure.

PBW(i)=∑j=120BW(j)×fi(j)
 MathType@MTEF@5@5@+=feaafiart1ev1aaatCvAUfKttLearuWrP9MDH5MBPbIqV92AaeXatLxBI9gBaebbnrfifHhDYfgasaacH8akY=wiFfYdH8Gipec8Eeeu0xXdbba9frFj0=OqFfea0dXdd9vqai=hGuQ8kuc9pgc9s8qqaq=dirpe0xb9q8qiLsFr0=vr0=vr0dc8meaabaqaciaacaGaaeqabaqabeGadaaakeaacqWGqbaudaWgaaWcbaGaemOqaiKaem4vaCfabeaakiabcIcaOiabdMgaPjabcMcaPiabg2da9maaqahabaGaemOqaiKaem4vaCLaeiikaGIaemOAaOMaeiykaKIaey41aqRaemOzay2aaSbaaSqaaiabdMgaPbqabaGccqGGOaakcqWGQbGAcqGGPaqkaSqaaiabdQgaQjabg2da9iabigdaXaqaaiabikdaYiabicdaWaqdcqGHris5aaaa@4936@

In Eq. 1, the index *j *runs over the 20 naturally occurring amino acids, *BW*(*j*) is the propensity value of amino acid *j *in the BW scale, and *f*_*i*_(*j*) the frequency of amino acid *j *in sequence position *i*. Plugging Eq. 1 into 0.5×(*C*(*i*) - *P*_*BW*_(*i*)), the overall approach of the BW method for deriving a positional score can be cast as follows.

S(i)=0.5×C(i)−∑j=1200.5×BW(j)×fi(j)
 MathType@MTEF@5@5@+=feaafiart1ev1aaatCvAUfKttLearuWrP9MDH5MBPbIqV92AaeXatLxBI9gBaebbnrfifHhDYfgasaacH8akY=wiFfYdH8Gipec8Eeeu0xXdbba9frFj0=OqFfea0dXdd9vqai=hGuQ8kuc9pgc9s8qqaq=dirpe0xb9q8qiLsFr0=vr0=vr0dc8meaabaqaciaacaGaaeqabaqabeGadaaakeaacqWGtbWucqGGOaakcqWGPbqAcqGGPaqkcqGH9aqpcqaIWaamcqGGUaGlcqaI1aqncqGHxdaTcqWGdbWqcqGGOaakcqWGPbqAcqGGPaqkcqGHsisldaaeWbqaaiabicdaWiabc6caUiabiwda1iabgEna0kabdkeacjabdEfaxjabcIcaOiabdQgaQjabcMcaPiabgEna0kabdAgaMnaaBaaaleaacqWGPbqAaeqaaOGaeiikaGIaemOAaOMaeiykaKcaleaacqWGQbGAcqGH9aqpcqaIXaqmaeaacqaIYaGmcqaIWaama0GaeyyeIuoaaaa@558D@

Eq. 2 indicates that *S*(*i*) is a linear combination of the conservation index and the 20 elements of the profile. Thus, it can be written more generally as follows.

S(i)=Cc×C(i)+∑j=120Cj×fi(j),
 MathType@MTEF@5@5@+=feaafiart1ev1aaatCvAUfKttLearuWrP9MDH5MBPbIqV92AaeXatLxBI9gBaebbnrfifHhDYfgasaacH8akY=wiFfYdH8Gipec8Eeeu0xXdbba9frFj0=OqFfea0dXdd9vqai=hGuQ8kuc9pgc9s8qqaq=dirpe0xb9q8qiLsFr0=vr0=vr0dc8meaabaqaciaacaGaaeqabaqabeGadaaakeaacqWGtbWucqGGOaakcqWGPbqAcqGGPaqkcqGH9aqpcqWGdbWqdaWgaaWcbaGaem4yamgabeaakiabgEna0kabdoeadjabcIcaOiabdMgaPjabcMcaPiabgUcaRmaaqahabaGaem4qam0aaSbaaSqaaiabdQgaQbqabaGccqGHxdaTcqWGMbGzdaWgaaWcbaGaemyAaKgabeaakiabcIcaOiabdQgaQjabcMcaPaWcbaGaemOAaOMaeyypa0JaeGymaedabaGaeGOmaiJaeGimaadaniabggHiLdGccqGGSaalaaa@4EA4@

where *C*_*c *_is the coefficient for the conservation index (set to 0.5 in the BW method) and *C*_*j *_the coefficient for the *j*th element of the profile (set to -0.5×*BW*(*j*) in the BW method). With achieving highest possible prediction accuracies in mind, we raise the question of whether setting the coefficients in Eq. 3 empirically as in the BW method is optimal or not. Our answer is no. Optimizing the coefficients would be a better idea. Confirming this expectation, the coefficients optimized by linear regression led to a prediction accuracy of 71.13%, compared to 68.67% of the BW method as shown in Table 1. Specifically, ridge linear regression with the complexity parameter set to 0.001 was used throughout this study in an effort to minimize generalization errors [50]. It is noteworthy that we use the same formula as the BW method – Eq. 3 – but with an entirely different philosophy. In the BW method, one first derives a propensity scale of the 20 amino acids to be exposed to the membrane from known HMP structures and then uses it for computing the propensity of a target residue to be exposed (Eq. 1). This propensity of the target residue is combined with its degree of conservation to yield its positional score. Our analysis reveals that this overall idea of the BW method can be concisely summarized by Eq. 3, which immediately suggests that there is a better way of doing the job.

**Table 1:** Prediction accuracies of different methods examined in the study

Prediction method	Prediction accuracy [%]^1^
The BW method	68.67
TMX	78.71
The YU method	71.06^2^

^1^Defined as the fraction of the TM residues in the data set whose burial status was correctly predicted.

^2^Best prediction accuracy among 16 ones shown in Table 6.

There is an issue to be clarified before we move on. We implemented the BW method, and its performance was evaluated on the same data set as for TMX. This was necessary since it is often difficult to directly compare performance values of different prediction methods reported in different studies because of the variety of data sets used and the discrepancy in state definitions. A serious difficulty arose in implementing the BW method, namely setting thresholds manually. As mentioned above, upon computing the positional score of a target residue, the BW method compares it with a threshold that has been manually set. If the positional score is greater than the threshold, it is predicted to be buried. Otherwise, it is predicted to be exposed. In a leave-one-out (jack-knife) testing scheme, thresholds need to be manually set separately for each of 43 protein chains in the benchmark data set (see Methods). Admittedly, it is impossible for us to exactly reproduce this step in the way it was performed in the original publication for the BW method [46]. In addition, we feel that it might be subjective to set thresholds manually. Then, is there any mathematical formalism that allows thresholds to be set in such a manner that (1) we exactly mimic the manual setting of thresholds as was done in the BW method and (2) yet, thresholds are set objectively and reproducibly? Our answer is a linear support vector classifier (lSVC, i.e. an SVC with a linear kernel). Since the hyperplane – *f*(*x*) = *β*_0 _+ *β*^T^*x *= 0, where *β*^T ^is the transpose of a column vector *β *– obtained by an lSVC in a one-dimensional space represents a scalar value of -*β*/*β*_0 _[50], setting a threshold via an lSVC is an exact computational analogue to setting it manually, yet in an objective, reproducible way. It is to be noted that the introduction of an lSVC to the prediction scheme transforms it to a two-step scheme because an lSVC also needs training and, as a result, the jack-knife scheme should be applied to both steps. We want to stress that the sole purpose of using an lSVC here is to mimic the manual assignment of thresholds as exactly as possible yet in an objective, reproducible fashion. Thus, we intentionally did not seek SVCs with a non-linear kernel or other sophisticated classifiers at this stage (but see below).

### Improved use of conservation indices

Another point well worth considering in Eq. 3 is how conservation indices are incorporated. The average identities of sequences retrieved from sequence databases for different query sequences can be appreciably varying. Thus, without normalization, one may assign overall high conservation indices to one protein chain while assigning overall low conservation indices to another. Normalization of conservation indices effectively solves this bias problem, just as in microarray data processing. In the BW method, conservation indices are not normalized. We found that normalizing conservation indices by subtracting the mean followed by division by the standard deviation separately for each protein chain leads to a significant improvement in the prediction accuracy, raising it from 71.13% to 73.84%.

A second, minor aspect to be considered is how conservation indices are actually computed in the first place. The BW method computes conservation indices as follows.

*C*(*i*) = 0.5 × *V*(*i*) + 0.5 × *IC*(*i*)

In Eq. 4, *V*(*i*) is the volume of the polytope for sequence position *i *derived from a multiple sequence alignment (MSA), estimating the probability for the presence of a set of different amino acids from a set of pairwise distribution probabilities, and *IC*(*i*) is the information content of sequence position *i *[46]. Eq. 4 relies on many assumptions that are yet to be validated. The first are ad hoc measures taken to enforce the Euclidean space to the distances between aligned sequences for computing *V*(*i*) [51]. The second is the assumption used in computing *IC*(*i*) that the 20 naturally occurring amino acids are equally likely to occur in the TM region. The third is that even though it seems reasonable to assign equal weights to both terms in Eq. 4, it is not clear whether that choice is optimal.

As in our previous studies [52,53], we derived conservation indices using Eq. 5, which is mathematically well-defined and relatively free from potentially problematic assumptions.

C(i)=∑j(fi(j)−f(j))2
 MathType@MTEF@5@5@+=feaafiart1ev1aaatCvAUfKttLearuWrP9MDH5MBPbIqV92AaeXatLxBI9gBaebbnrfifHhDYfgasaacH8akY=wiFfYdH8Gipec8Eeeu0xXdbba9frFj0=OqFfea0dXdd9vqai=hGuQ8kuc9pgc9s8qqaq=dirpe0xb9q8qiLsFr0=vr0=vr0dc8meaabaqaciaacaGaaeqabaqabeGadaaakeaacqWGdbWqcqGGOaakcqWGPbqAcqGGPaqkcqGH9aqpdaGcaaqaamaaqababaGaeiikaGIaemOzay2aaSbaaSqaaiabdMgaPbqabaGccqGGOaakcqWGQbGAcqGGPaqkcqGHsislcqWGMbGzcqGGOaakcqWGQbGAcqGGPaqkcqGGPaqkdaahaaWcbeqaaiabikdaYaaaaeaacqWGQbGAaeqaniabggHiLdaaleqaaaaa@4335@

In Eq. 5, the index *j *runs over the 20 naturally occurring amino acids, *C*(*i*) is the conservation index for sequence position *i*, *f*_*i*_(*j*) is the frequency of amino acid *j *in sequence position *i*, and *f*(*j*) is the overall frequency of amino acid *j *in the alignment. As expected, the use of Eq. 5 instead of Eq. 4 improved the prediction accuracy from 73.84% to 74.51%. It is to be noted that conservation indices obtained by Eqs. 4 and 5 were from the same MSAs.

### Extending the window size for the input vector

At this stage, the input vector for the prediction method consists of 21 elements (20 profile elements and a conservation index for the target residue). Another measure that we can take to further improve the prediction accuracy is to additionally consider the neighboring residues of the target residue (i.e. increasing a window size for the input vector from 1 to any larger number). In fact, nearly all techniques developed for water-soluble proteins exploit this possibility [11-26]. We explored all symmetric window sizes (Table 2). There are a couple of points to be noted in Table 2. When increasing the window size from 1 to 3 or 5, the prediction accuracy is decreased, suggesting that the signal-to-noise ratio deteriorated (see also below). The first peak in the prediction accuracy is observed at a window size of 9. It is interesting to note that, assuming the canonical helix conformation, when the length of a helix gets to 9, the first and last residues (residues at positions *i*-4 and *i*+4) face in the same direction as the central residue (residue at position *i*, corresponding to the target residue in our context). Thus, our results suggest that the identities of the residues lying just above and below the target residue on the same helix face are most indicative of the burial status of the target residue, as expected from the canonical helix conformation. As it is actually 3.6 residues per turn in the canonical helix conformation, a certain improvement is already found by including the positions *i *± 3. Consistent with this line of reasoning, the best prediction accuracy, 75.97%, is observed at a window size of 15. Based on a similar observation, Adamian and Liang recently developed a highly effective method for predicting membrane-exposed faces of TM helices [47].

**Table 2:** Prediction accuracies obtained by linear regression with different window sizes

Window size	Prediction accuracy
1	74.51
3	73.55
5	73.96
7	74.82
9	75.69
11	75.37
13	75.81
15	75.97
17	75.46
19	75.75
21	75.59

### Feature selection

The logic behind increasing window sizes for better predictions is that one can better account for long-range effects with enlarged windows. However, the shortcoming of enlarged windows is that the signal-to-noise ratio deteriorates as the window size is increased, as demonstrated in Table 2. For example, compare the prediction accuracies for window sizes of 15 and 21. The tradeoff between long-range effects and signal-to-noise ratios would suggest a window size of 15 instead of 21. Is there any way of circumventing this unpleasant tradeoff? Feature selection might be an answer. A simple illustration will make this point clearer. An input vector for a window size of 21 consists of 441 elements (21 elements for each of the 21 residues). It is intuitively clear that not all 441 elements will contribute equally to the prediction. Many of them might simply be noise. Thus, it might be possible to use enlarged windows for a better consideration of long-range effects and still maintain a high signal-to-noise ratio by filtering out noisy elements.

Of many techniques available for feature selection, we chose the Fisher's index for the following reasons. First, the Fisher's index is conceptually attractive, having a clear meaning easy to understand [50]. Put simply, the Fisher's index represents the ability of a given element to maximize the distance between the centroids of the two given classes and simultaneously minimize the overlap between them. Second, unlike techniques involving linear combinations of feature vectors, the Fisher's index is highly interpretable. This is a big advantage given the high dimensions of our feature spaces. Most importantly, one can gain interesting biological insights into the structural organization of HMPs from the Fisher's index (see below). Third, the Fisher's index can be computed cheaply. Fourth, the Fisher's index is well suited to continuous features (as opposed to discrete ones).

The 441 elements of a window of size 21 were ranked according to their Fisher's indices, and increasing fractions of them (in steps of 0.05) were input to the prediction (see first and second columns of Table 3). The best prediction accuracy, 77.21%, was obtained when using the top 20% elements only. This accuracy is higher than 75.97% obtained by an "unintelligently" increased window of size 15 in the above section. Which elements rank top? As shown in Table 4, the top-ranking elements are mostly conservation indices, in line with previous findings that conservation properties of TM residues correlate strongly with their degree of exposure to the membrane [52,54-56]. Also, Table 4 shows that the frequencies of occurrence of L, I, V and F at the target residue are highly indicative of its burial status. In this regard, it is interesting to note that our previous study showed that these amino acids possess the highest propensities to preferentially interact with the membrane [53]. The frequency of occurrence of G at the target residue is also strongly correlating with its burial status, ranking at the 9th place, which is consistent with earlier findings that glycine residues play a pivotal role in mediating helix-helix interactions in the membrane [57-60]. Table 4 also shows that the frequencies of occurrence of I, G and L at the 4th residue N terminal to the target one also strongly correlate with the burial status of the target residue, which makes sense considering the canonical helix conformation as mentioned above.

Given dramatic improvements in prediction accuracy and interesting insights into the system under investigation through a feature selection as demonstrated here, it was quite surprising to find that almost all studies on predicting the solvent accessibility of water-soluble proteins [11-24] have not considered it. Hence, it would be worthwhile to investigate whether feature selection can similarly pay off in predicting the solvent-accessibility of water-soluble proteins.

**Table 3:** Prediction accuracies obtained by increasing fractions of the 441 elements of a window of size 21

Fraction used in the prediction	Linear regression	SVR C – 10^1^	SVR C – 1	SVR C – 0.1	SVR C – 0.01
0.05^2^	75.65	70.36	73.45	75.21	74.89
0.1	76.61	71.06	75.88	75.97	74.57
0.15	76.90	70.78	75.11	76.20	74.09
0.2	77.21	70.65	75.14	75.78	73.58
0.25	76.45	70.01	75.59	75.78	73.04
0.3	76.04	71.13	75.11	75.24	72.56
0.35	75.72	71.54	74.79	75.27	71.54
0.4	75.91	72.69	74.76	75.11	71.80
0.45	75.91	72.72	75.11	74.95	71.86
0.5	76.13	72.82	75.33	75.11	71.67
0.55	76.39	72.63	75.43	75.43	72.08
0.6	76.04	72.69	75.43	75.14	71.61
0.65	75.33	72.94	75.24	75.30	70.40
0.7	74.86	73.01	74.98	74.92	70.24
0.75	75.33	73.20	75.43	74.86	69.31
0.8	75.75	72.75	75.75	74.44	68.48
0.85	75.62	72.59	75.75	74.22	67.97
0.9	75.24	72.34	75.14	74.00	67.65
0.95	75.21	72.79	75.46	74.22	67.53
1.0	75.59	73.26	75.97	74.16	67.85

^1^Regularization constant C was set to 10.

^2^Meaning that the top 5% of the 441 elements when ranked by the Fisher's index were input for the prediction.

### Non-linear regression

All approaches for computing positional scores thus far can be understood as an extension of Eq. 3. Namely, they are all linear methods. Additional improvements might be achieved by relying on non-linear methods. The power of non-linearity is illustrated by conservation indices. Conservation indices are non-linear combinations of profile elements (Eq. 5), which was motivated by the prior knowledge that conserved TM residues tend to be buried while variable ones tend to be exposed to the membrane [52,54-56]. In fact, Table 4 showed that conservation indices were the features most strongly correlated with the burial status of TM residues. Also, it is shown below that conservation indices play a much greater role than profile elements in boosting prediction accuracies. In theory, a perfect non-linear method should be able to find such non-linear combinations of profile elements when fed only profile elements. However, this is usually not the case. Whenever prior knowledge on the system under investigation permits sensible non-linear combinations of raw features (e.g., conservation indices out of profile elements), it is always good to do so explicitly.

**Table 4:** Top 20 elements of the 441 ones of a window of size 21 according to the Fisher's index

Rank	Position^1^	Type	Fisher's index
1	T	conservation index	0.987
2	C4	conservation index	0.534
3	N4	conservation index	0.469
4	N3	conservation index	0.307
5	N7	conservation index	0.306
6	C3	conservation index	0.248
7	T	L^2^	0.243
8	C7	conservation index	0.240
9	T	G	0.203
10	T	I	0.143
11	C8	conservation index	0.132
12	C1	conservation index	0.092
13	T	V	0.059
14	N1	conservation index	0.057
15	N4	I	0.057
16	N4	G	0.056
17	T	F	0.053
18	T	S	0.052
19	N8	conservation index	0.045
20	N4	L	0.041

^1^T: the target residue, C4: the 4th residue C terminal to the target residue, N4: the 4th residue N terminal to the target residue. Thus, the conservation index of the target residue is most indicative of its burial status, and the conservation index of the 4th residue C terminal to the target residue is second most indicative of the burial status of the target residue.

^2^L: leucine, G: glycine, I: isoleucine, V: valine, F: phenylalanine and S: serine.

If there still remain untapped non-linear combinations of profile elements or profile elements and conservation indices that correlate with the burial status of TM residues, the use of non-linear methods might be profitable [61]. Of the vast array of available non-linear regression techniques, we made use of SVR with a radial kernel because a nice interface with R is already available (see Methods) and it has performed respectably in studies of water-soluble proteins [23,25]. Our preliminary analysis showed that SVR with a radial kernel tends to rival SVR with other kernels. Once a kernel type is chosen, another important parameter to be fine-tuned is the regularization constant C, i.e. how much weight one should put on minimizing the costs of violating a decision boundary relative to maximizing the closest distance of a data point to the boundary [50,62]. The general expectation from the theory is that as the regularization constant gets higher, a heavier weight is put on minimizing the violation costs and, as a result, a more wiggly decision boundary is obtained with a possibly larger generalization error. The default C value is 1, and we tried 4 different C values, 10, 1, 0.1 and 0.01. As above, the 441 elements of a window of size 21 were ranked according to their Fisher's indices, and increasing fractions of them (in steps of 0.05) were input to the prediction via SVR with a radial kernel. Table 3 shows the results (columns 3 – 6). It is immediately clear that, in almost all cases, linear regression outperforms SVR, indicating that the generalization errors of SVR are larger than those of linear regression, presumably due to its over-flexibility in fitting a separating boundary to a given data set. Thus, SVR does not seem advantageous over linear regression on this data set. Admittedly, we can not rule out the possibility that highly fine-tuned SVR can outperform linear regression. Given limited computational resources and considerable amounts of computation required for a leave-one-out validation of a two-step prediction method (~40 CPU hours on a 2.4 GHz processor), it is beyond our capability to exhaustively scan all possible combinations of SVR parameters. However, it is our experience that SVR with all parameters set to default values generally performs nearly optimally. Thus, we are quite certain that, at least for the current purpose of predicting the burial status of TM residues of HMPs, linear regression is at least as effective as SVR. Supporting this conclusion, previous studies on water-soluble proteins demonstrated that sophisticated linear methods can rival non-linear ones in performance [14,21,26].

### Optimizing classifiers

Upon computing a positional score for the target residue, a classifier is invoked to classify it as either buried in the protein core or exposed to the membrane. Although any machine-learning technique can be used as a classifier, we have only considered lSVCs so far. The original reason for choosing lSVCs was, as mentioned earlier, to implement the BW method as exactly as possible, yet in an objective, reproducible manner, so that the BW method can be justly compared with ours. However, we may choose other classifiers for our prediction method. Although there are tons of available classifiers, we primarily focused on SVCs for practical reasons as mentioned above. Preliminary analysis showed that SVCs with a linear or radial kernel tend to outperform others. Thus, SVCs with a linear or radial kernel were pursued further in combination with 5 different regularization constants, 1, 0.5, 0.1, 0.05 and 0.01, chosen on the basis of the results shown in Table 3. In addition to searching for a better classifier, it might also be helpful in boosting prediction accuracies to refine input vectors themselves. So far, the input vectors for a classifier have been one-dimensional, i.e. consisting of a positional score for a given target residue. The input vectors for a classifier can be straightforwardly refined exactly in the same way as the input vectors for computing positional scores were refined in Table 3.

Table 5 shows the best prediction accuracies for each combination of an SVC kernel and a regularization constant. An SVC with a linear kernel outperforms that with a radial one, and a regularization constant of 0.5 is optimal among those investigated. The best prediction accuracy, 78.71%, was obtained by an SVC with a linear kernel that considers the top 16 positional scores out of the 21 positional scores (i.e. the positional scores of the target residue and its 10 neighbors on the N terminus and its 10 neighbors on the C terminus) derived from considering the top 10% of the 441 elements of a window of size 21 (Table 3). An SVC with a radial kernel also achieved this prediction accuracy at a regularization constant of 0.5. Due to its sustained performance over the examined range of regularization constants, however, an SVC with a linear kernel is preferred. The method that gives rise to the best performance becomes the method of choice and is named "TMX (TM eXposure)." Detailed jack-knife test results of TMX are available as additional information [see Additional file 1] and also on its web server.

The performance of TMX – is it "significantly" higher than that of the BW method? As mentioned earlier, the prediction accuracy of the BW method is 68.67% when tested on the same data set. The *p *value estimating the statistical significance of the 10.04% increase in the prediction accuracy achieved by TMX relative to the BW method is < 10^-5 ^according to the Wilcoxon signed rank test. Accordingly, TMX is judged to be a truly better method for predicting the burial status of TM residues of HMPs. A final point worthy of noting is the architecture of TMX. TMX is a two-step prediction method, where binary classifications are made in the second step on the basis of positional scores computed in the first step. The two-step architecture – is it really worthwhile? Obviously, one can directly apply SVCs to the profiles and conservation indices of the target residue and its neighbors for predicting its burial status, without computing positional scores in the first place. Several studies on water-soluble proteins noted that a two-step prediction scheme can better account for correlated patterns of properties to be predicted, leading to higher prediction accuracies [7-10,21,23,24]. To test whether this is also the case for us, we investigated the performance of SVCs that were directly fed profiles and conservation indices for the prediction. Specifically, as shown in Table 3, the 441 elements of a window of size 21 were sorted according to the Fisher's index, and increasing fractions of them were fed to SVCs. The best prediction accuracy for an SVC with a linear kernel was 77.53%, and that for an SVC with a radial kernel 77.21%. Therefore, a two-step prediction scheme appears to pay off in our case, too.

**Table 5:** Best prediction accuracies for each combination of an SVC kernel and a regularization constant C

	Regularization constant C				
Kernel	1	0.5	0.1	0.05	0.01
Linear	78.62	78.71	78.65	78.62	78.01
Radial	78.55	78.71	78.23	78.30	77.25

### Comparison with the YU method

The YU method computes the positional score of a target residue via SVR using position-specific scoring matrices (PSSMs) obtained by PSI-BLAST [63]. In studies of water-soluble proteins, it has been very popular to use PSSMs as input vectors in order to boost the accuracy of predicting solvent accessibility [8-10,17,19-24]. The popularity of PSSMs has partially stemmed from the fact that one does not have to explicitly generate an MSA for obtaining PSSMs. As with the BW method, we implemented the YU method for a transparent performance comparison using the R interface [64,65] of the LIBSVM library [66]. In implementing the YU method, we set all the parameters of SVR as optimized by Yuan *et al*. and did not intentionally seek any further optimizations.

The best prediction accuracy of the YU method on the benchmark data set is 71.06% (fourth column of Table 6), much lower than 78.71% achieved by TMX (*p *value of < 0.001 from the Wilcoxon signed rank test). It is of interest to find out where the performance difference between TMX and the YU method comes from, except for the novelties introduced to TMX such as feature selection and a sophisticated classification in the second step. To this end, we replaced PSSMs by profiles or conservation indices to find out how different input vectors affect prediction accuracies. Table 6 shows that profiles alone perform similarly to (or only slightly better than) PSSMs. Compared with the performance of profiles or PSSMs, the performance of normalized conservation indices is really standing out. Moreover, a comparison of the performance of profiles plus normalized conservation indices shown in Table 2 with that of normalized conservation indices alone (C set to 1, a default value, in Table 6) also indicates that conservation indices play a crucial role in boosting prediction accuracies. Thus, it may be concluded that the poor performance of the YU method is partly due to the fact that its input vectors – PSSMs – do not contain the information captured by conservation indices. In this regard, it is interesting to note that the most effective method for predicting the solvent accessibility of water-soluble proteins uses PSSMs as its sole input [24]. Thus, it would be worthwhile to check out whether replacing PSSMs by profiles plus normalized conservation indices would be similarly successful for water-soluble proteins.

**Table 6:** Prediction accuracies obtained by SVR with different input vectors

Window size	Profile	Conservation index	PSSM (the YU method)
C – 1^1^			

11	70.87	73.68	70.08
13	71.16	73.65	69.47
15	71.51	74.16	71.06
17	71.19	74.16	68.23
19	70.91	74.41	61.85
21	70.84	74.19	59.46

C – 2			

11	70.55	73.17	69.85
13	70.94	73.26	69.31
15	71.16	74.31	70.52
17	71.64	73.61	67.11
19	70.68	73.90	61.54
21	70.49	74.09	59.31

C – 5			

11	70.36	72.37	69.79
13	71.19	72.53	69.12
15	70.91	73.36	70.43
17	71.32	72.72	66.92
19	70.46	73.07	61.60
21	70.59	72.85	59.18

C – 7			

11	70.81	72.05	70.17
13	71.03	72.15	69.15
15	71.13	72.94	70.43
17	71.19	72.37	66.89
19	70.43	72.50	61.63
21	70.52	72.08	59.18

^1^Regularization constant C was set to 1.

### Analysis of the TMX predictions

In addition to prediction accuracies, there are other interesting aspects worthy of analyzing. For example, are there any amino acids for which it is easier to predict the burial status? Is it easier to correctly predict buried residues as being buried than exposed residues as being exposed?

Table 7 shows the results for each amino acid. The highest prediction accuracies were achieved for R, H, D and K, all of which are charged or strongly polar. Their average conservation indices are among the highest (data not shown). Thus, it appears that these amino acids are well conserved for functional (and/or structural) reasons and that their high conservation indices make it easier for TMX to correctly predict their burial status. In this regard, the case of proline is a contrasting example. Its average conservation index is among the highest, yet the prediction accuracy for it is among the lowest. The data set contains 43 buried and 46 exposed proline residues, and the average conservation indices for buried and exposed proline residues are 1.22 and 1.07, respectively. Thus, proline residues exposed to the membrane appear as strongly conserved for their structural (and/or functional) role as those buried inside. The lack of correlation between conservation and the burial status for proline seems to make it difficult for TMX to correctly predict its burial status. Surprisingly, E is the amino acid with the lowest prediction accuracy. Inspection of the individual incorrect predictions for E suggests a couple of plausible explanations for this unexpected result. First, conserved E residues are sometimes exposed to the membrane (2GIF_A_346: 1.54 [residue 346 of chain A in the PDB file 2GIF: its conservation index is 1.54], 1OTS_A_414: 2.46, 1YEW_B_201: 1.43 and 2BL2_A_139: 3.47), and TMX predicted them to be buried. Second, there are several buried E residues that are not conserved (2A65_A_112: -0.46, 2A65_A_419: -0.94, 1SU4_A_908: -0.30 and 1QLA_C_180: 0.14), and presumably their low conservation indices hinder the accurate prediction of their burial status. The prediction accuracies for abundant amino acids (L, A, V, I, G and F) are all higher than the overall accuracy of 78.71%.

**Table 7:** Prediction accuracies for each amino acid

Amino acid	Number of occurrence	Prediction accuracy [%]	Fraction of exposed residues in the data set [%]
A	381	80.31	45.93
C	50	74.00	46.00
D	19	89.47	0.00
E	30	56.67	40.00
F	294	80.95	73.13
G	316	80.38	27.22
H	42	90.48	16.67
I	328	80.49	72.26
K	20	85.00	55.00
L	521	80.42	73.70
M	128	75.78	57.03
N	41	75.61	17.07
P	89	61.80	48.31
Q	26	76.92	26.92
R	15	100.00	6.67
S	153	71.24	39.22
T	169	73.37	48.52
V	365	79.45	65.48
W	74	81.08	70.27
Y	77	72.73	50.65

Table 8 shows the specificity and sensitivity of TMX. The lower sensitivity (70.61%) compared to the specificity (84.77%) seems to reflect the biased composition of the benchmark data set comprising 55.86% of exposed residues and 44.14% of buried residues.

**Table 8:** Specificity and sensitivity of TMX

	Observed	
	
Predicted	Buried	Exposed
Buried	978 (70.61%)	267 (15.23%)
Exposed	407 (29.39%)	1486 (84.77%)

Sum	1385	1753

### Confidence scores for the predictions made

**Figure 1:**
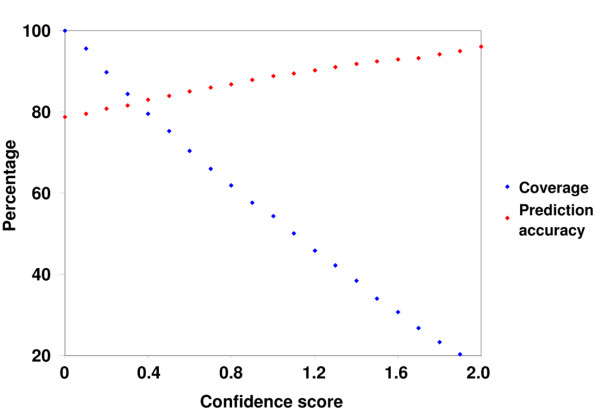
Prediction accuracy and coverage depending on confidence scores. When considering all predictions (i.e. predictions with a confidence score ≥ 0.00), the prediction accuracy is 78.71% and the coverage is 100%. When considering only the 1440 predictions with a confidence score ≥ 1.20, the prediction accuracy rises to 90.21% and the coverage falls down to 45.89%.

It is highly desirable to have confidence scores available for the predictions made. Confidence scores allow the user to selectively utilize prediction results in real application settings. In TMX, the classification is performed using an SVC. It is intuitive that predictions made by an SVC with a high decision value (i.e. a large distance to the decision boundary) would be more accurate, and we found out that this is indeed the case. Thus, the absolute magnitude of a decision value generated by the SVC is taken to be a confidence score for the prediction [50,62]. As shown in Fig. 1, predictions with a high confidence score tend to be more accurate than those with a low one. The prediction accuracy rises to 90.21% when considering the 1440 predictions with a confidence score ≥ 1.2. 1440 out of 3138 means a coverage of 45.89%. Thus, a fairly high coverage is maintained for prediction accuracies of ~90%, which makes TMX well suited to real application settings.

### Outlook

In this study, we have focused on HMPs because they are much more abundant and play a more important role in diverse cellular processes than beta-barrel membrane proteins (BMPs). But, obviously, one can apply the same strategy behind the development of TMX to BMPs. Since the burial status of TM residues of BMPs tends to alternate due to prevalent beta strand elements [67], it would be interesting to see whether one can achieve higher prediction accuracies than 78.71% for HMPs. Moreover, it would be worth investigating which features correlate strongly with the burial status of TM residues of BMPs via a feature selection of the sort used here for Table 4.

## Conclusion

We have presented TMX, a novel sequence-based computational method for predicting the burial status of TM residues of HMPs. It significantly outperforms previously proposed methods. In addition, feature selection incorporated in TMX revealed interesting insights into the structural organization of HMPs. Importantly, unlike the previous methods, TMX automatically generates confidence scores for the predictions made, and it was shown that predictions with a high confidence score tend to be more accurate than those with a low one. Thus, in a real application setting, the user of TMX can selectively utilize prediction results on the basis of their confidence scores. The developmental course of TMX clearly highlighted the importance of conservation indices and feature selection in boosting prediction accuracies. In this regard, it was rather surprising to find that the most effective method for predicting the solvent accessibility of water-soluble proteins considers neither conservation indices nor feature selection. It would be interesting to investigate whether these two "new" findings can be favorably transferred to one of the classical bioinformatics problems of predicting the solvent accessibility of water-soluble proteins.

## Methods

### Generation of the benchmark data set

As is always the case in machine-learning studies, constructing a well-curated data set was the starting point of the current study. Special care was taken in selecting protein chains, delineating their TM boundaries and computing the rSASA values of TM residues.

The details of generating a non-redundant high-quality data set have been described elsewhere [53]. Briefly, based on the lists of HMPs with known structure compiled by White [68] and by Michel [69] as of February 2007, protein chains with less than 25% pairwise identity and a resolution better than 3.0 Å were gathered, resulting in 43 protein chains of 3138 TM residues (Table 9). Predictions were made using full-length protein sequences. Yet the benchmark analysis reported here was limited to those residues in the hydrophobic core of the membrane as defined in the OPM database [70] because the binary burial status is meaningful only for them. 51 TM residues were further excluded in the analysis because they are mostly located in the N or C terminus and thus no alignment of homologous sequences could be made (see below). The data set available for download on the TMX web server comprises 3138 TM residues included in the benchmark analysis, which includes not only TM helices but also fragments of re-entrant regions that dip into the hydrophobic core of the membrane. The classification of a residue as being exposed vs. buried was based on its rSASA value. To approximate the effective radius of the CH_2 _group of hydrocarbon chains of phospholipids, the probe radius was set to 2.2 Å. When necessary, the two faces of the TM region (the cytoplasmic and exoplasmic faces) were capped with dummy atoms before computing SASA values. Many HMPs contain large internal cavities, and, without capping, large SASA values were assigned to residues lining internal cavities, making these residues look as if they were facing the membrane. Upon capping, internal cavities that are inaccessible to the probe were identified and excluded in computing SASA values. Actual computations were carried out using the program suite VOLBL [71,72]. SASA values were normalized by dividing them by reference values to yield rSASA values. The reference value for an amino acid, X, is its SASA in the context of a nonapeptide helix GGGG-X-GGGG computed with a probe radius of 2.2 Å as above.

**Table 9:** 43 Protein chains used in the study

PDB ID	Protein	Chains
1. 1M0L	Bacteriorhodopsin	A
2. 1GZM	Rhodopsin	A
3. 1R3J	KcsA potassium channel	C
4. 1J4N	Aquaporin	A
5. 1LDF	Glycerol facilitator channel	A
6. 1XQF	Ammonia channel	A
7. 1OTS	H^+^/Cl^- ^exchanger	A
8. 2A65	Leucine transporter	A
9. 2CFQ	Lactose permease	A
10. 1YEW	Methane monooxygenase	B, C
11. 1SU4	Calcium ATPase	A
12. 2BL2	Rotor of V-type Na^+^-ATPase	A
13. 1DXR	Photosynthetic reaction center	L, M, H
14. 1KF6	Fumarate reductase (*E. coli*)	C, D
15. 1QLA	Fumarate reductase (*W. succinogenes*)	C
16. 1KQF	Formate dehydrogenase N	B, C
17. 1Q16	Nitrate reductase A	C
18. 1NEK	Succinate dehydrogenase	C, D
19. 1ZOY	Complex II	C, D
20. 1OKC	Mitochondrial ADP/ATP carrier	A
21. 1V55	Cytochrome C oxidase (aa_3 _type)	B, D, G, I, J, L, M
22. 1EHK	Cytochrome C oxidase (ba_3 _type)	A, B
23. 1PP9	Cytochrome bc_1 _complex	D, E, G, J
24. 2GIF	AcrB multidrug efflux transporter	A
25. 2IC8	GlpG rhomboid-family intramembrane protease	A
26. 2NQ2	Putative metal-chelate-type ABC transporter	A

Exposed residues were defined as those with an rSASA greater than 0.00, as in a previous study [73]. This threshold rSASA value is justified for HMPs given the large probe radius chosen in this study. As discussed before [25], this threshold is also free from artifacts arising from normalization and subsequent binary classification. Nevertheless, it was argued that the threshold for a binary classification should be set such that the data set is equally partitioned into the two classes to avoid statistical artifacts. An rSASA of 0.00 induces a slightly skewed partitioning of 44.14% of buried residues and 55.86% of exposed ones. Equipartitioning of the data set was achieved with an rSASA of 0.04. Additional analysis showed that the conclusions drawn in this study remain fully valid for this new threshold (data not shown).

### Computation of profiles and conservation indices

In general, the use of a profile (the frequencies of the 20 amino acids for a sequence position) improves the performance of sequence-based prediction methods. For extracting profiles, one needs to generate MSAs. As with any sequence-based prediction methods, the careful choice of sequences in MSAs is very important for the performance of the prediction method. MSAs generated using different criteria would yield results of differing quality. Thus, it would be desirable to generate "optimal" MSAs for different query sequences. Unfortunately, it is currently impossible to do so in an objective, consistent manner without any prior knowledge about the three-dimensional structures of the query sequences. Thus, a reasonable approach that is also objective, consistent and easily reproducible by others, was taken for generating MSAs, even though it might produce suboptimal MSAs for some query sequences. Its detail has been described elsewhere [52,53]. Briefly, for a given query sequence, a maximum of 1000 homologous sequences were retrieved from the non-redundant database using BLAST [63]. Initial MSAs were built using ClustalW [74]. Then, sequence fragments were deleted from the MSA. Sequences that are less than 25% identical to the query sequence were also removed. The remaining sequences were realigned using ClustalW to yield a final MSA, which was used to obtain profiles. When deriving profiles from an MSA, amino acid frequencies were weighted using a modified method of Henikoff and Henikoff as implemented in PSI-BLAST [63,75]. Actual computations were performed using the program AL2CO [76]. Conservation indices (Eq. 5) were also derived using AL2CO.

### Support vector machines

The support vector classifier (SVC)/support vector regression (SVR) [50,62] implementation in R [64-66] was used for the current work. The parameters for SVC/SVR were set to default values unless otherwise noted. The YU method was implemented using the SVR implementation in R as described in [49].

### Performance evaluation

A leave-one-out ('jack-knife') test was carried out to measure the performance of different prediction methods examined in this study. For two-step prediction methods, the jack-knife scheme was applied to both steps as it should be. Prediction accuracies mean the fractions of the benchmark data set for which the burial status was correctly predicted.

## List of abbreviations used

HMP: helical membrane protein, TM: transmembrane, SVC: support vector classifier, lSVC: SVC with a linear kernel, SVR: support vector regression, MSA: multiple sequence alignment

## Authors' contributions

YP designed the study, and YP, SH and VH performed the study and prepared the manuscript.

## Supplementary Material

Additional file 1 Detailed jack-knife test results. The data shows the detailed jack-knife test results of TMX. Click here for file
