# Supplementary material for: Prediction of the burial status of transmembrane residues of helical membrane proteins
Source: BMC Bioinformatics. 2007 Aug 20;8:302. doi: 10.1186/1471-2105-8-302 (PMC2000914; doi:10.1186/1471-2105-8-302)
Supplement: Additional file 1 — Detailed jack-knife test results. The data shows the detailed jack-knife test results of TMX. [file 1471-2105-8-302-S1.pdf]

Detailed jack-knife test results

C1	C2	C3	C4	C5	C6	C7	C8
2nq2	A	143	114	79.72	2995	2348	78.40
2ic8	A	94	74	78.72	3044	2397	78.75
1m0l	A	96	77	80.21	3042	2398	78.83
1gzm	A	112	89	79.46	3026	2371	78.35
1r3j	C	42	39	92.86	3096	2432	78.55
1j4n	A	111	96	86.49	3027	2372	78.36
1ldf	A	104	93	89.42	3034	2368	78.05
1xqf	A	160	129	80.62	2978	2336	78.44
1ots	A	184	153	83.15	2954	2308	78.13
2a65	A	171	120	70.18	2967	2339	78.83
2cfq	A	194	147	75.77	2944	2324	78.94
1yew	B	76	51	67.11	3062	2407	78.61
1yew	C	49	42	85.71	3089	2415	78.18
1dxr	L	79	60	75.95	3059	2406	78.65
1dxr	M	83	61	73.49	3055	2405	78.72
1dxr	H	15	11	73.33	3123	2456	78.64
1su4	A	152	114	75.00	2986	2350	78.70
2bl2	A	65	49	75.38	3073	2424	78.88
1kf6	C	45	31	68.89	3093	2436	78.76
1kf6	D	56	40	71.43	3082	2436	79.04
1qla	C	77	65	84.42	3061	2402	78.47
1kqf	B	18	18	100.00	3120	2451	78.56
1kqf	C	78	66	84.62	3060	2407	78.66
1nek	C	50	37	74.00	3088	2427	78.59
1nek	D	50	42	84.00	3088	2433	78.79
1zoy	C	52	44	84.62	3086	2427	78.65
1zoy	D	39	33	84.62	3099	2443	78.83
1q16	C	89	68	76.40	3049	2397	78.62
1okc	A	105	81	77.14	3033	2361	77.84
1v55	B	24	17	70.83	3114	2447	78.58
1v55	D	14	11	78.57	3124	2465	78.91
1v55	G	16	14	87.50	3122	2450	78.48
1v55	I	11	9	81.82	3127	2454	78.48
1v55	J	14	12	85.71	3124	2457	78.65
1v55	L	13	10	76.92	3125	2451	78.43
1v55	M	13	10	76.92	3125	2454	78.53
1ehk	A	212	160	75.47	2926	2314	79.08
1ehk	B	15	13	86.67	3123	2457	78.67
1pp9	D	13	8	61.54	3125	2462	78.78
1pp9	E	13	12	92.31	3125	2465	78.88
1pp9	G	13	10	76.92	3125	2463	78.82
1pp9	J	13	7	53.85	3125	2465	78.88
2gif	A	165	133	80.61	2973	2325	78.20

C1: PDB ID
C2: Chain ID
C3: Number of TM residues considered
C4: Number of TM residues correctly predicted
C5: C4*100/C3, test prediction accuracy in percentage (jack-knife test prediction accuracy)
C6: Number of training data
C7: Number of training data correctly predicted
C8: C7*100/C6, training prediction accuracy in percentage
